# *DkPK* Genes Promote Natural Deastringency in C-PCNA Persimmon by Up-regulating *DkPDC* and *DkADH* Expression

**DOI:** 10.3389/fpls.2017.00149

**Published:** 2017-02-13

**Authors:** Changfei Guan, Xiaoyun Du, Qinglin Zhang, Fengwang Ma, Zhengrong Luo, Yong Yang

**Affiliations:** ^1^State Key Laboratory of Crop Stress Biology for Arid Areas, College of Horticulture, Northwest A&F UniversityYangling, China; ^2^Key Laboratory of Horticultural Plant Biology – Ministry of Education, Huazhong Agricultural UniversityWuhan, China; ^3^Institute of Pomology, Yantai Academy of Agricultural SciencesYantai, China

**Keywords:** persimmon, PAs, *DkPK*, *DkPDC*, *DkADH*, qRT-PCR, transient over-expression, deastringency

## Abstract

The astringency of Chinese pollination-constant non-astringent (C-PCNA) persimmon (*Diospyros kaki* Thunb.) can be naturally removed on the tree. This process is controlled by a single locus and is dominant against other types of persimmons; therefore, this variant is an important candidate for commercial cultivation and the breeding of PCNA cultivars. In our previous study, six full-length coding sequences (CDS) for pyruvate kinase genes (*DkPK1-6*) were isolated, and *DkPK1* is thought to be involved in the natural deastringency of C-PCNA persimmon fruit. Here, we characterize the eight other *DkPK* genes (*DkPK7-14*) from C-PCNA persimmon fruit based on transcriptome data. The transcript changes in *DkPK7-14* genes and correlations with the proanthocyanidin (PA) content were investigated during different fruit development stages in C-PCNA, J-PCNA, and non-PCNA persimmon; *DkPK7* and *DkPK8* exhibited up-regulation patterns during the last developmental stage in C-PCNA persimmon that was negatively correlated with the decrease in soluble PAs. Phylogenetic analysis and subcellular localization analysis revealed that DkPK7 and DkPK8 are cytosolic proteins. Notably, *DkPK7* and *DkPK8* were ubiquitously expressed in various persimmon organs and abundantly up-regulated in seeds. Furthermore, transient over-expression of *DkPK7* and *DkPK8* in persimmon leaves led to a significant decrease in the content of soluble PAs but a significant increase in the expression levels of the pyruvate decarboxylase (*DkPDC*) and alcohol dehydrogenase genes (*DkADH*), which are closely related to acetaldehyde metabolism. The accumulated acetaldehyde that results from the up-regulation of the *DkPDC* and *DkADH* genes can combine with soluble PAs to form insoluble PAs, resulting in the removal of astringency from persimmon fruit. Thus, we suggest that both *DkPK7* and *DkPK8* are likely to be involved in natural deastringency via the up-regulation of *DkPDC* and *DkADH* expression during the last developmental stage in C-PCNA persimmon.

## Introduction

Persimmon (*Diospyros kaki* Thunb.), which is native to China, is the most economically important species of the genus *Diospyros* and is mainly cultivated in China, Japan, and Korea. Currently, cultivation of this crop is rapidly expanding with fruit growers in Italy, Spain, and Israel (Yamada, 2004; Yonemori et al., 2008). Persimmon varieties can be categorized into two groups according to genetic traits and natural deastringency: pollination-constant non-astringent (PCNA) persimmons, including Chinese PCNA (C-PCNA) and Japanese PCNA (J-PCNA) persimmons, and non-PCNA types, including pollination-constant astringent (PCA), pollination-variant non-astringent (PVNA), and pollination-variant astringent (PVA) types (Akagi et al., 2011). Most non-PCNA fruits accumulate large amounts of high molecular weight proanthocyanidins (PAs) that cause a strong astringency sensation in fresh fruits; therefore, these fruits are often undesirable for human consumption before artificial treatment for astringency removal. PCNA persimmon fruits are able to remove astringency naturally during the ripening stage, thus justifying their significant value as commercial products. Furthermore, C-PCNA and J-PCNA differ in their genetic characteristics of natural deastringency: C-PCNA is controlled by a single locus and is dominant against J-PCNA (Ikegami et al., 2004, 2006). Therefore, C-PCNA-type persimmons hold great potential for breeding new PCNA cultivars. However, the mechanism of natural deastringency in the C-PCNA type is not very clear.

Astringency removal from persimmon fruits has long been a topic of traditional research, including deastringency treatments for non-PCNA persimmon fruits and natural deastringency of PCNA-type persimmon. Condensed tannins, also known as PAs, are colorless phenolic polymers that accumulate in vacuoles (Yakushiji and Nakatsuka, 2007). This accumulation causes the astringency in the persimmon fruit. In non-PCNA persimmon, artificial deastringency treatment can promote the synthesis of acetaldehyde. The accumulation of acetaldehyde in persimmon fruit is thought to be directly involved in the coagulation of soluble PAs, resulting in a loss of astringency (Taira, 1995; Salvador et al., 2007). The acetaldehyde metabolism-related genes pyruvate decarboxylase (*PDC*) and alcohol dehydrogenase (*ADH*) are key elements involved in astringency removal from ‘Mopanshi’ (non-PCNA) persimmon (Min et al., 2012). The expression of *PDC* and *ADH* increase significantly in non-PCNA persimmon fruits during deastringency treatment with ethanol, CO_2_, or warm water (Min et al., 2012; Luo et al., 2014; Guan et al., 2015). In J-PCNA type persimmon, the growth of PAs in cells ends during the early developmental stages of persimmon fruit, with the loss of astringency principally occurring via PA dilution as the fruit grows larger (Yonemori et al., 2003). It has been reported that PA biosynthesis is predominantly controlled by the DkMyb4 transcription factor in J-PCNA persimmon, in which the down-regulation of *DkMyb4* expression during the early stages of fruit growth leads to a substantial down-regulation of PA pathway genes, which results in a reduction in PA accumulation (Akagi et al., 2009). Compared with J-PCNA and non-PCNA types, the mechanisms of astringency removal in C-PCNA persimmon fruit are not well understood. Astringency removal in the C-PCNA persimmon fruit is hypothesized to possess the characteristics of both the J-PCNA and non-PCNA types: the astringency removal is related to the dilution effect and to PA insolubilization (the conversion of soluble tannins into insoluble tannins) (Zhang et al., 2012; Mo et al., 2016). Several studies have suggested that acetaldehyde-mediated PA coagulation occurring during the late stages of fruit growth is likely to play a more important role in deastringency in the C-PCNA persimmon (Zhang et al., 2012; Mo et al., 2016).

Acetaldehyde metabolism-related genes *ADH*, *PDC*, and pyruvate kinase (*PK*) are considered key candidates for involvement in the natural removal of astringency during the last developmental stage in C-PCNA persimmon (Guan et al., 2016; Mo et al., 2016). *PDC*, a gene downstream of *PK*, catalyzes the generation of acetaldehyde from pyruvate. ADH catalyzes the reversible reaction between acetaldehyde and ethanol. The *DkADH1*, *DkPDC1*, and *DkPDC2* genes were up-regulated specifically at late stages of C-PCNA fruit development, during which soluble PA contents decrease rapidly as a result of the coagulation of soluble PAs into insoluble PAs (Guan et al., 2016; Mo et al., 2016). Transient over-expression of *DkADH1* and *DkPDC2* could lead to a significant decrease in the content of soluble PAs in persimmon leaves (Mo et al., 2016). Furthermore, in our previous study, transient over-expression of *DkPK1* in persimmon leaves resulted in not only a significant decrease in the content of soluble PAs, but a significant increase in the levels of *DkADH1*, *DkADH3*, *DkPDC1*, *DkPDC3*, *DkPDC4*, and *DkPDC5* transcripts (Guan et al., 2016). PK, the upstream enzyme of acetaldehyde metabolism, is a key regulatory enzyme of the glycolytic pathway that catalyzes the irreversible conversion of PEP and ADP to pyruvate and ATP. There are abundant studies showing that the reaction is a primary control site of plant glycolytic flux toward pyruvate (Plaxton, 1996). Only one form of PK is believed to exist in most bacteria and lower eukaryotes, although two forms of PK have been found in *Escherichia coli* (Malcovati et al., 1973; Muñoz and Ponce, 2003). In contrast with non-plant PKs, both cytosolic PK (PKc) and plastid PK (PKp) isozymes exist in plants, and these types show pronounced differences in their respective physical and kinetic/regulatory characteristics (Plaxton, 1996; Plaxton et al., 2002). PKp has been shown to be critical for providing pyruvate and ATP to several plastidic biosynthetic pathways, such as the accumulation of oil in seed (Knowles et al., 1998; Andre et al., 2007; Oliver et al., 2008), whereas PKc function is more variable due to its involvement in a range of biosynthetic pathways as well as respiration (Knowles et al., 1998; Oliver et al., 2008). It has been reported that PKc plays an important role in responding to defense-related abiotic stresses in *Capsicum annuum* (Kim et al., 2006), in regulating pyruvate and alternative oxidase levels in heterotrophic tissues in potato (Oliver et al., 2008) and in regulating fruit sugar content during the ripening period in loquat fruit (Qin et al., 2013). Thus, considering the roles of *PK*c in plants and the previous study of *DkPK1* involvement in the natural deastringency, the PK cytosolic protein family is more likely to be an important contributor to the natural removal of astringency in C-PCNA persimmon. Although we have isolated and characterized full-length cDNAs for six *DkPK* genes in our previous study (Guan et al., 2016), other members of the *DkPK* family and their relationship with natural deastringency remain to be further explored.

In the present research, full-length cDNAs of the *DkPK7-10* genes (Accession Nos. KY292502 to KY292509) were isolated from ‘Luotian-tianshi’ persimmon fruit based on transcriptome data (Luo et al., 2014). Next, the expression levels of *DkPK* genes and their correlations with soluble PAs were analyzed at different developmental stages of C-PCNA, J-PCNA, and non-PCNA persimmon fruits. Furthermore, subcellular localization and transient over-expression were used to confirm the function of candidate *DkPK* genes in persimmon leaves. Finally, the effect of over-expressing the *DkPK* genes in persimmon leaves on the expression of the *DkPDC* and *DkADH* genes are discussed. Our study will not only be helpful for understanding the mechanism of astringency removal and but will also contribute to the breeding of PCNA cultivars in the future.

## Materials and Methods

### Materials

**FIGURE 1:**
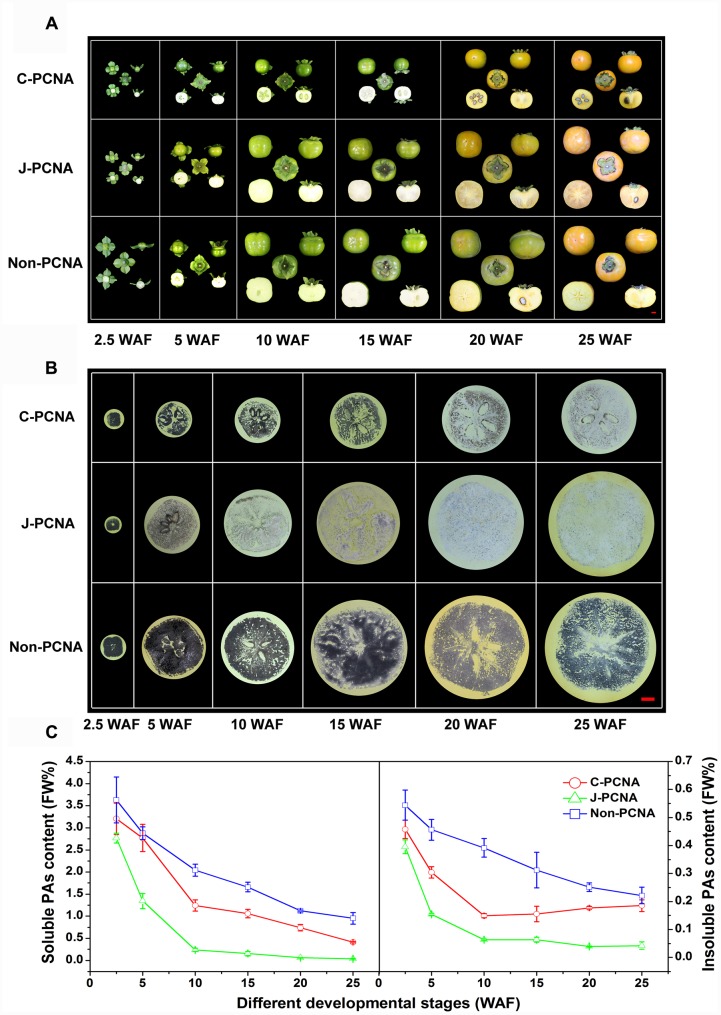
**Representative photos of fruits and measurement of PA content in three types of persimmon during fruit development. (A)** Photographs showing the fruits sampled at six stages: 2.5, 5, 10, 15, 20, and 25 WAF of C-PCNA, J-PCNA, and non-PCNA persimmon. **(B)** Analysis of soluble PA content using an imprinting method. **(C)** Analysis of soluble and insoluble PA content using the Folin–Ciocalteu method. Flesh was sampled at 2.5, 5, 10, 15, 20, and 25 WAF. FW%, PA concentration per fresh weight; WAF, weeks after flowering. The three photographs of ‘Mopanshi’ persimmon fruits at 25 WAF and ‘Luotian-tianshi’ persimmon fruits at 20 and 25 WAF were from Mo et al. (2016).

Fruits of three types of persimmons (*D*. *kaki* Thunb.; 2*n* = 6x = 90) from ‘Luotian-tianshi’ (C-PCNA), ‘Youhou’ (J-PCNA) and ‘Mopanshi’ (non-PCNA) were sampled from the Persimmon Repository, Huazhong Agricultural University, Wuhan, China. Fruit flesh from uniform fruits were sampled at 2.5, 5, 10, 15, 20, and 25 weeks after flowering (WAF) (**Figure 1A**). Leaf, stem, sepal, peel, pulp, seed, and core samples were collected for tissue-specific gene expression at 22 WAF, and flowers were harvested at full flowering stage. All the samples were immediately frozen in liquid nitrogen and stored at -80°C. All treatments were performed with three biological replicates of 12 fruits each.

### PA Content Analysis

Insoluble and soluble PAs were measured using the Folin–Ciocalteu method (Oshida et al., 1996), with absorbance measured at 725 nm using a UV-2450 spectrophotometer (Shimadzu, Japan). In addition, the printing method (Eaks, 1967) was used to determine the soluble PA content, e.g., darker the filter paper indicates that more soluble PAs are present.

### RNA Extraction and cDNA Synthesis

Total RNA was isolated from frozen flesh samples using the RNAplant Plus Reagent (Tiangen Biotech Co., Beijing, China). A NanoDrop 2000 spectrophotometer (Thermo Fisher Scientific, Waltham, MA, USA) and gel electrophoresis were used for detection of the RNA quality and quantity. Three biological replicates were performed for each sample. First-strand cDNA for gene isolation was generated according to the manufacturer’s protocol for the EasyScript One-Step gDNA Removal and cDNA Synthesis SuperMix Kit (TransGen, Beijing, China). The cDNA for gene expression was synthesized using a PrimeScript RT Kit with gDNA Eraser (TaKaRa, Dalian, China).

### Gene Isolation and Sequence Analysis

Other members of the *DkPK* family were isolated based on the C-PCNA persimmon RNA-Seq data we reported previously (Luo et al., 2014). Full-length cDNAs of *PK* genes were amplified with a SMART RACE cDNA Amplification Kit (Clontech, USA). The sequences of the primers used for RACE and cloning are described in Supplementary Table S1. The gene sequences were translated with online software^1^ and confirmed with BLAST methods in GenBank. Deduced amino acid sequences of homologous genes in other species were retrieved from Oliver et al. (2008) and NCBI. A phylogenetic tree was constructed with MEGA6 software (Tamura et al., 2011) with the neighbor-joining (NJ) method. Major motifs of DkPK proteins were identified using the MEME^2^ and MOTIF Search^3^ online tools.

### Quantitative Reverse Transcription PCR (qRT-PCR)

qRT-PCR was performed with the ABI One Step Plus Real-Time PCR System (Applied Biosystems, Carlsbad, CA, USA). The PCR reaction mixture (20 μl total volume) included 10 μl SYBR^®^ Premix Ex Taq^TM^ II (Tli RNaseH Plus) (TaKaRa, Dalian, China), and 7.0 μl sterile water, 1.0 μl diluted cDNA, 0.8 μl each primer (10 μM) and ROX Reference Dye 0.4 μl. The standard PCR conditions of three steps were as follows: 3 min at 95°C, then 45 cycles of 95°C for 5 s, 58°C for 30 s, and 72°C for 30 s. *DkActin* (Akagi et al., 2009) was set as the internal reference, each sample was analyzed in quadruplicate, and all primers are listed in Supplementary Table S1.

### Subcellular Localization

To determine the subcellular localization of DkPK7 and DkPK8, the complete open reading frame (ORF) without the termination codon was amplified using their respective primers (Supplementary Table S1). The construction of fusion vectors 35S-DkPK7/8::YFP was performed according to our previous report (Guan et al., 2016). The 35S-YFP was used as a control. The fusion and control plasmids were transiently transferred into the leaves of 6-week-old *Nicotiana benthamiana* plants via *Agrobacterium*-mediated transformation. The *p19* gene from tomato bushy stunt virus transformed together with the fusion construct was used to suppress *DkPK7/8* gene silencing (Grefen et al., 2008). Two days later, the fluorescence signal was observed through a confocal laser scanning microscope (CLSM, Olympus Fluoview FV1000, Japan).

### Transient Transformation in Persimmon Leaves

A transient over-expression system was constructed to analyze the function of *DkPK* genes in the coagulation of soluble PAs in persimmon leaves *in vivo*. The full-length coding region of *DkPK7/8* was amplified by reverse transcription PCR (RT-PCR) with their respective primers (Supplementary Table S1). The construction and transient transformation analysis of the PMV2-DkPK fusion vectors were performed according to our reports (Mo et al., 2015; Guan et al., 2016). pMV2-GFP was set as the control vector. On the eighth day, 0.1 g tissue from each over-expressing leaf was collected for the measurement of PA content, with a total of 12 single-leaf replicates.

## Results

### PA Content Analysis

The imprinting method was first used to examine soluble PA content in C-PCNA, non-PCNA, and J-PCNA persimmons fruits (**Figure 1B**). In J-PCNA persimmon, the reaction elicited an obvious color change from dark before 5 WAF to increasingly pale after 10 WAF, indicating that the soluble PA concentration decreased sharply and remained at low levels (0.24–0.04%) until the last developmental stage. Compared with the J-PCNA persimmon, the soluble PAs in non-PCNA persimmon fruit remained at a high level throughout all stages of development; the consistently high level demonstrates that natural deastringency does not occur naturally in non-PCNA persimmon. It is worth noting that the reaction exhibited a pale color for fresh C-PCNA persimmon fruit at the last developmental stage, suggesting that the concentration of soluble PAs decreased to a low level during astringency removal.

To confirm the imprinting results, the Folin–Ciocalteu method was performed for quantitative determination of soluble and insoluble PA contents (**Figure 1C**). During the entire developmental process, the level of soluble PAs in C-PCNA persimmon fruit was higher than that in the J-PCNA type, whereas it was lower than that of the non-PCNA type; these results showed a pattern similar to that seen with the imprinting method (**Figure 1B**). The soluble PA content in the three persimmons all showed significant decreases, indicating that a dilution effect of soluble PAs was shared among different types of persimmon as the fruits grow larger. Both soluble and insoluble PA contents in J-PCNA persimmon decreased significantly from 2.5 to 10 WAF, and the low level was maintained during the last developmental stage (10–25 WAF); this trend was consistent with the previous report showing that the growth of tannin cells stops during the early developmental stage and results in the natural deastringency of J-PCNA persimmon fruit (Akagi et al., 2009). In contrast, the non-PCNA cultivar still had a high PA content even at the last developmental stage and retained its astringency at 25 WAF. Compared with the non-PCNA type, the soluble PA content of C-PCNA persimmon decreased rapidly from 15 to 25 WAF; this decrease led to the natural removal of astringency. At the same time, the insoluble PA content exhibited the opposite pattern, and it accumulated rapidly. These results were consistent with previous reports that the coagulation of soluble PAs during the last developmental stage might cause natural deastringency in C-PCNA persimmon fruit (Zhang et al., 2012; Guan et al., 2016; Mo et al., 2016).

### Gene Isolation and Sequence Analysis

**FIGURE 2:**
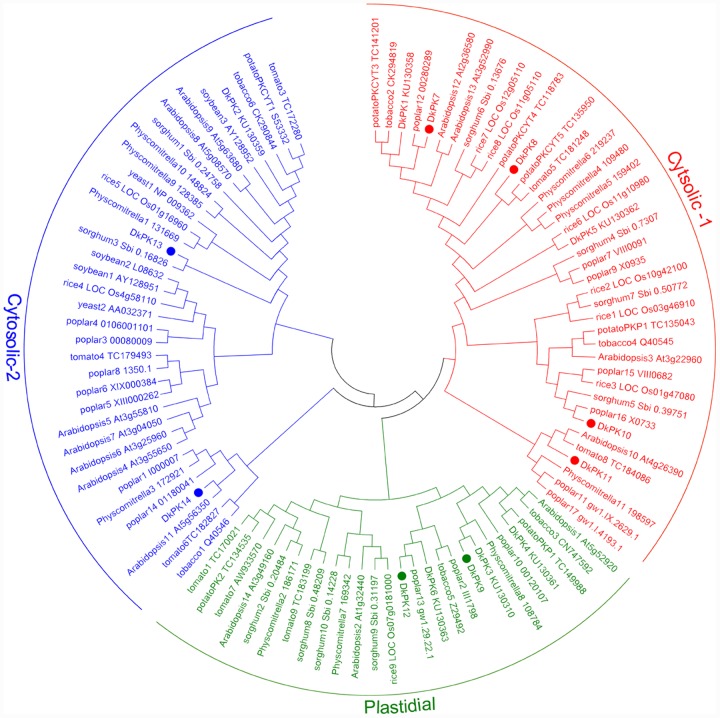
**Phylogenetic analysis of deduced DkPK amino acid sequences**. DkPK proteins from other species were obtained from NCBI and Oliver et al. (2008); the deduced amino acid sequences of DkPK1-6 were obtained from Guan et al. (2016). The persimmon PK7-14 proteins are marked with filled circles. The phylogenetic tree was constructed with MEGA6.

**FIGURE 3:**
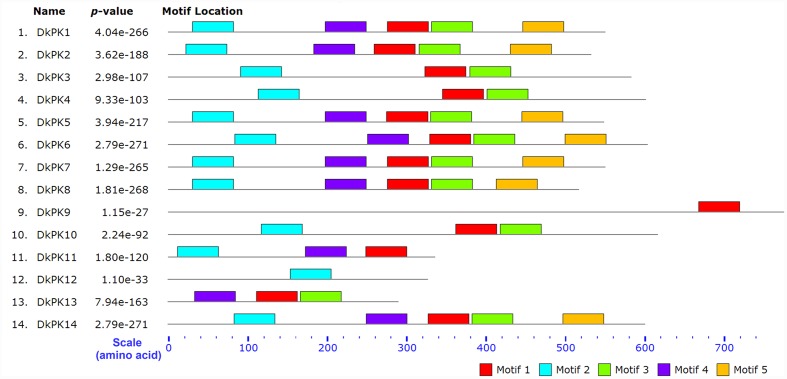
**Motif distribution of deduced DkPK amino acid sequences**. The lengths and positions of motifs in the protein sequences were indicated using colored blocks. The barrel domain: motif 1, motif 2, and motif 4; the RibD C-terminal domain: motif 3; the alpha/beta domain: motif 5. The deduced amino acid sequences of DkPK1-6 were obtained from Guan et al. (2016). Major motifs were identified using the MEME and MOTIF Search online tools.

The cDNAs of eight *DkPK* genes (*DkPK7-14*) were isolated from C-PCNA persimmon fruit based on cDNA-SSAP and RNA-seq data. Of these genes, *DkPK7-10* were full-length coding sequences (CDS), while the others were partial CDS. To evaluate sequence divergences and identify conserved motifs, phylogenetic analysis and the MEME tool were used to analyze the deduced amino acid sequences of *DkPK* genes. Phylogenetic analysis of the deduced amino acid sequences showed that the plastidial and cytosolic PK isozymes were divided into two groups, and the latter contained two subgroups: cytosolic-1 and cytosolic-2 (**Figure 2**). DkPK7, DkPK8, DkPK10, and DkPK11 belonged to the cytosolic-1 subfamily, DkPK9 and DkPK12 clustered together in the plastidial group, and DkPK13 and DkPK14 were classified into the cytosolic-2 subgroup. DkPK7 clustered close to poplar12 and DkPK1, DkPK8 was close to potatoPKCYT5 and tomato5, DkPK10 was similar to poplar16, and DkPK11 was similar to tomato8 and arabidopsis10 (**Figure 2**). The determination of the putative cellular localization of DkPK7-14 is also supported by a grant from the WoLF PSORT program (Horton et al., 2007). The five major motifs were identified using the MEME and MOTIF Search online tools (**Figure 3**). Motif 1, motif 2, and motif 4 were the barrel domain; motif 3 belonged the RibD C-terminal domain; motif 5 was the alpha/beta domain of pyruvate kinase. Motif 1 was shared in all sequences, indicating that the barrel domain is essential for DkPK proteins. Motif 5 was often present together with the other four motifs in the cytosolic proteins of DkPK1-2, DkPK5-8, and DkPK14 predicted by phylogenetic analysis. Some motifs, such as motif 2, were present in most sequences. In addition, multiple sequence alignment of the DkPK proteins showed that they all shared the typical PK domain and had the 72.52% sequence identity in **Supplementary Figure S1**.

### Transcription Analysis of *DkPK7-14* during Fruit Development in C-PCNA, J-PCNA, and Non-PCNA Persimmon

**FIGURE 4:**
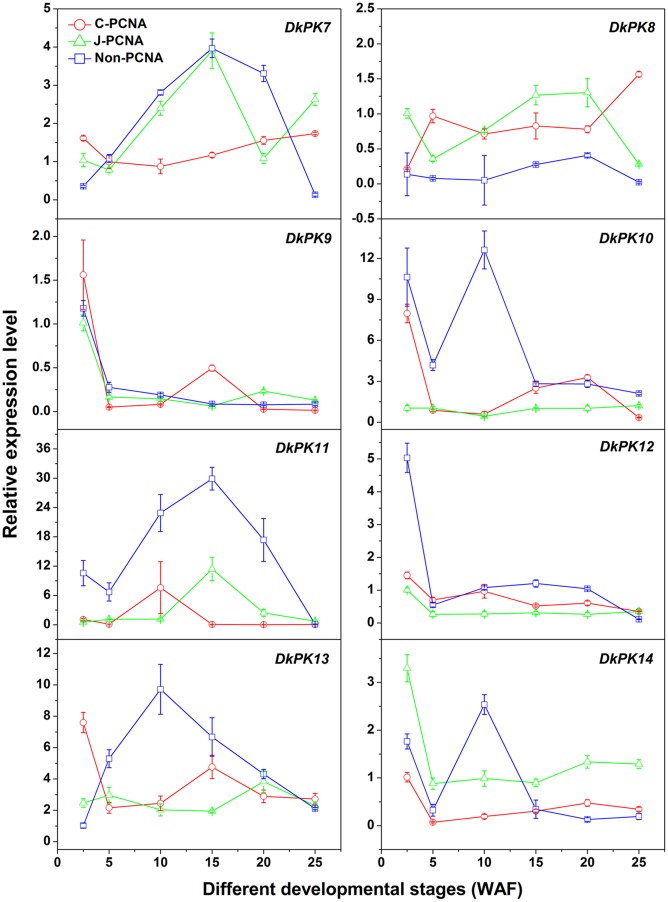
**Transcript changes in *DkPK7-14* in three types of persimmon during fruit development**. Flesh was sampled at 2.5, 5, 10, 15, 20, and 25 WAF. WAF, weeks after flowering. *Error bars* represent the standard deviation (*n* = 3).

To evaluate the relationships between *PK7-14* gene transcription and the PA content in C-PCNA persimmon, qRT-PCR analysis was performed at different C-PCNA persimmon developmental stages (**Figure 4**). J-PCNA and non-PCNA cultivars were used as controls. It is worth noting that *DkPK7* expression showed a trend that was similar to the changes in insoluble PAs in the C-PCNA type throughout development, with the transcript level decreasing rapidly from 2.5 to 10 WAF and then increasing gradually until the last stage. Additionally, *DkPK8* expression in the C-PCNA type increased rapidly during the last stage (20–25 WAF); this trend was in opposition to the soluble PA content at the same stage. Conversely, the expression of *DkPK8* in both J-PCNA and non-PCNA persimmon fruit was comparatively constant at the last stage. The transcript abundance of *DkPK9* and *DkPK13* in C-PCNA persimmon peaked at 2.5 WAF, increased from 5 to 15 WAF, and then remained relatively constant during the last stage. *DkPK10*, *DkPK12*, and *DkPK14* expression in the C-PCNA type showed a decreasing trend during the last stage, while the RNA transcripts of *DkPK10* and *DkPK12* in J-PCNA persimmon were relatively stable. The level of *DkPK11* expression in C-PCNA and J-PCNA peaked at 10 and 15 WAF, respectively, showing a trend of increasing during early stages of development and then decreasing during later stages. Because the *DkPK7* and *DkPK8* expression pattern, which exhibited an increasing trend during the last developmental stage and reached the highest level at 25 WAF, correlated with the PA content at the same developmental stage, we chose *DkPK7* and *DkPK8* as candidates for further analysis of their role in natural deastringency in C-PCNA persimmon.

### DkPK7 and DkPK8 Are Localized in the Cytoplasm of Tobacco Leaf Cells

**FIGURE 5:**
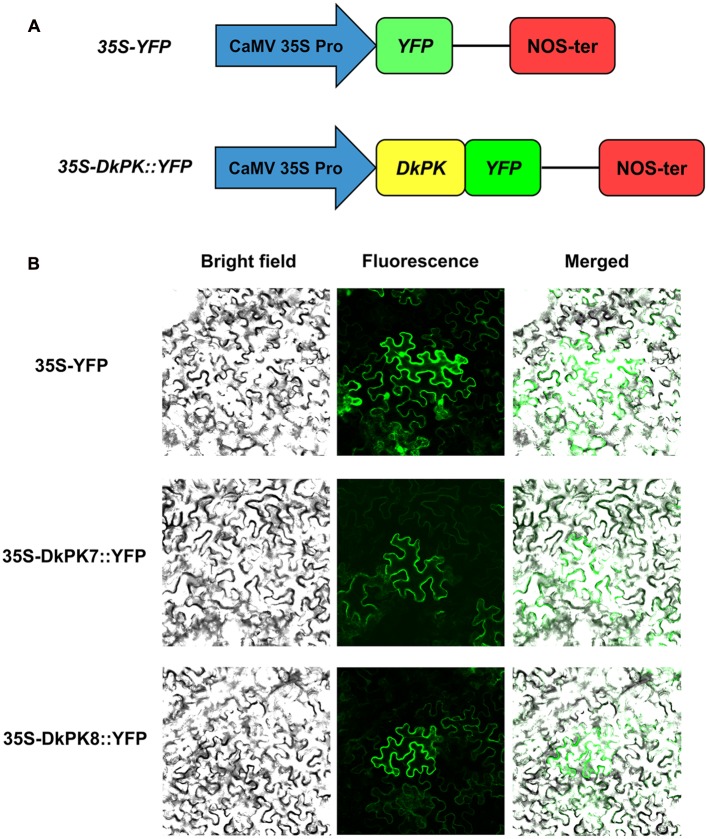
**Subcellular localization of the DkPK7 and DkPK8 fusion proteins in *Nicotiana benthamiana* leaf epidermal cells. (A)** Schematic diagram of the control *35S-YFP* and the *35S-DkPK::YFP* fusion genes. **(B)** The 35S-DkPK7::YFP and 35S-DkPK8::YFP fusion vectors and the 35S-YFP negative control were transiently transformed into tobacco leaf epidermal cells together with the *p19* gene via the agroinfiltration method.

To further confirm the prediction from phylogenetic analysis that DkPK7 and DkPK8 are located in the cytoplasm, subcellular localization was performed in tobacco leaf cells (**Figure 5**). The 35S-DkPK7::YFP and 35S-DkPK8::YFP fusion constructs and the 35S-YFP negative control were transiently transformed into *Nicotiana benthamiana* leaf cells together with the *p19* gene using the agroinfiltration method. Co-infiltration with the tomato bushy stunt virus silencing suppressor P19 can enhance the expression of various proteins in plants (Voinnet et al., 2003). Significantly, fluorescence signals were observed only in the cytoplasm when 35S-DkPK7::YFP and 35S-DkPK8::YFP were transfected, whereas the transient expression of 35S-YFP was detected in both the cytoplasm and nucleus (**Figure 5B**). This result demonstrated that DkPK7 and DkPK8 are cytosolic proteins and is in agreement with the prediction that DkPK7 and DkPK8 belong to the cytosolic family of proteins.

### *DkPK7* and *DkPK8* Were Highly Expressed in the Seeds of C-PCNA Persimmon

**FIGURE 6:**
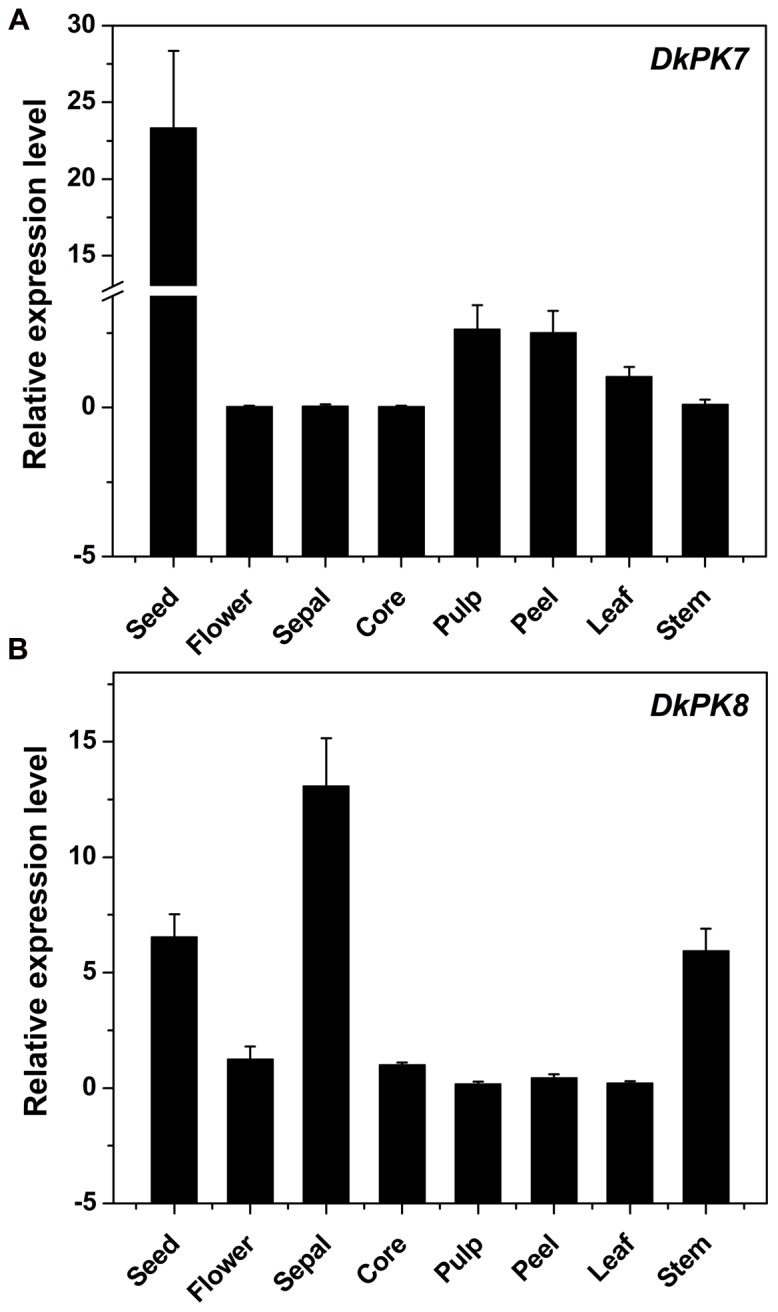
**Transcript analysis of *DKPK7* (A)** and *DKPK8* **(B)** in different tissues from C-PCNA persimmon. Seed, sepal, core, pulp, peel, leaf and stem from C-PCNA persimmon were sampled at 22 WAF; flower tissue was sampled at the full-bloom stage. *Error bars* represent the standard deviation (*n* = 3).

The *DkPK7* and *DkPK8* expression patterns were evaluated in different tissues, including seed, flower, sepal, core, pulp, peel, leaf, and stem, from the C-PCNA persimmon (**Figure 6**). Expression of *DkPK7* and *DkPK8* can be detected in various persimmon organs, with the highest expression of *DkPK7* in the seed (at least 10 times higher than that in the other tissues) and the highest expression of *DkPK8* in the sepal. mRNA expression of *DkPK8* was present at an elevated level in the seed, followed by the stem, in C-PCNA persimmon. This result was in agreement with our previous report that *DkPK1* was abundantly expressed in the seed of C-PCNA persimmon, and the presence of *DkPK1* is thought to contribute to the natural removal of astringency in C-PCNA persimmon (Guan et al., 2016). Furthermore, previous studies have reported that the seeds of some non-PCNA and C-PCNA persimmon can accumulate a volume of volatile acetaldehyde, which accelerates the coagulation of soluble PAs (Sugiura and Tomana, 1983; Mo et al., 2016). Thus, we speculate that the highly transcribed genes *DkPK7* and *DkPK8* in seeds might contribute to natural deastringency in C-PCNA persimmon on some level.

### Over-Expression of *DkPK7* and *DkPK8* Decreased the Soluble PA Content in Persimmon Leaves *In vivo*

**FIGURE 7:**
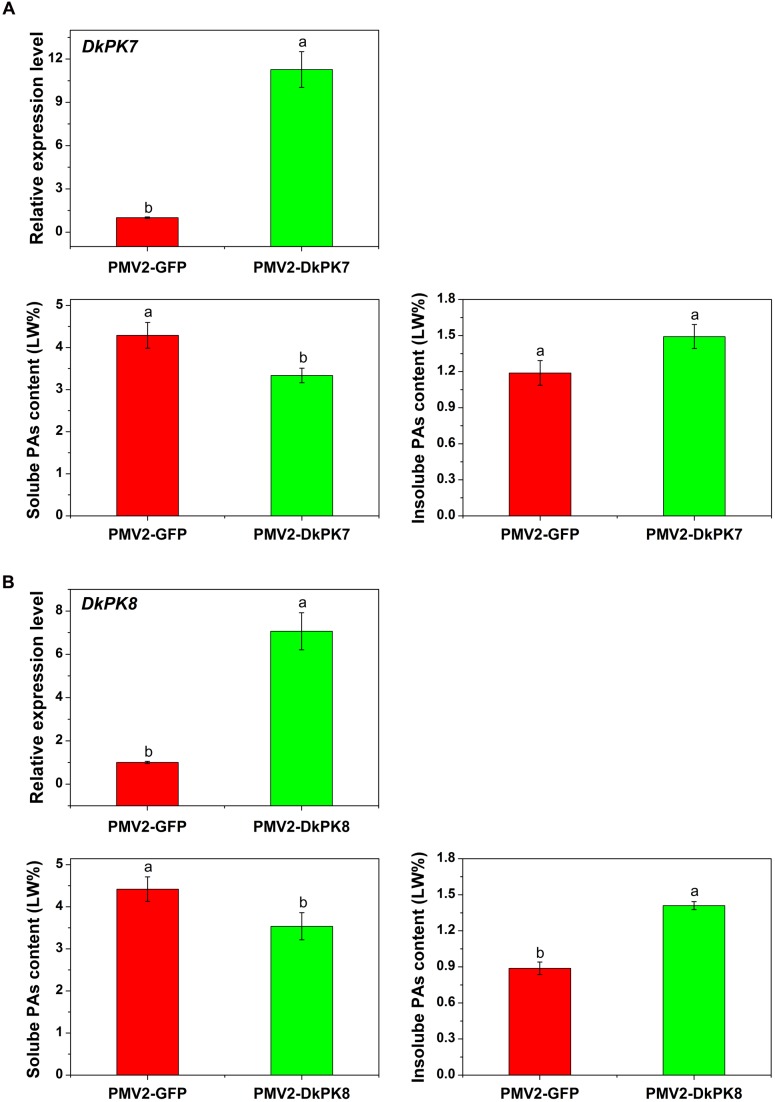
**Transient over-expression of *DKPK7* and *DKPK8* in persimmon leaves *in vivo*. (A)** Analysis of *DkPK7* transcript level, soluble PA and insoluble PA content in *DkPK7*-overexpressing leaves. **(B)** Analysis of *DkPK8* transcript level, soluble PA and insoluble PA content in *DkPK8*-overexpressing leaves. Leaves infiltrated with *DKPK7* and *DKPK8* in ‘Mopanshi’ persimmon were sampled at 8 days after agroinfiltration. pMV2-GFP indicates the empty vector. *Error bars* represent the standard deviation (*n* = 12) (*p* < 0.05).

To confirm the putative role of *DkPK*s in the solidification of soluble PAs *in vivo*, a transient-expression system was chosen for rapid gene functional analysis in persimmon leaves (**Figure 7**). The full-length CDS of the *DkPK* genes were fused to the PMV2 vector to generate the PMV2-DkPK constructs, and then, the constructs were put into persimmon leaves *in vivo* using an *Agrobacterium*-mediated transient transformation system (Mo et al., 2015). The PMV2-GFP fusion construct was used as a control. Both the *DkPK7* and *DkPK8* genes were transfected into and expressed successfully in persimmon leaves *in vivo*. The levels of *DkPK7* and *DkPK8* transcripts were increased by 11.3- and 7.1-fold, respectively, compared with the control in the infiltrated leaves. Furthermore, soluble PA content showed a significant decrease in persimmon leaves infiltrated with both *DkPK7* and *DkPK8* (**Figure 7**). In contrast, the level of insoluble PAs was increased in both transfected leaves, though there was not a significant difference in the *DkPK7*-over-expressing leaves; this tendency was the opposite of that of soluble PA. These results demonstrated that over-expression of *DkPK7* and *DkPK8* could lead to a remarkable (*p* < 0.05) reduction in the content of soluble PA, but a rise in the level of insoluble PA, in persimmon leaves *in vivo*.

### Over-Expression of *DkPK7* and *DkPK8* Enhanced the *DkPDC* and *DkADH* Transcript Levels in Persimmon Leaves *In vivo*

**FIGURE 8:**
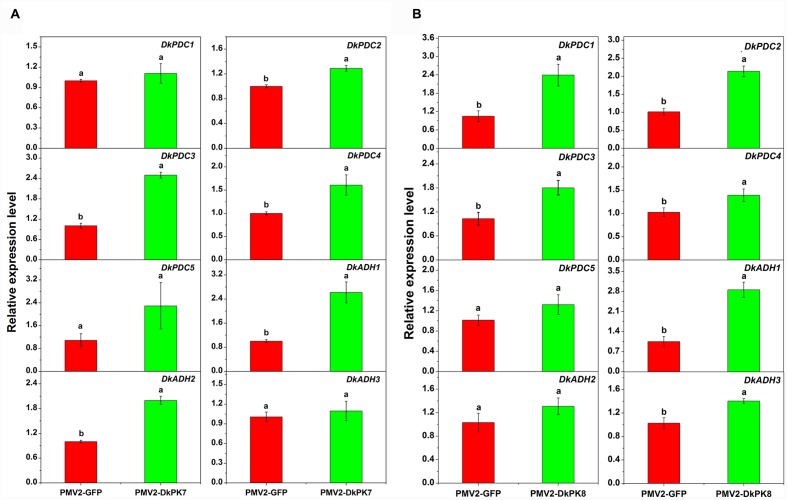
**Transient over-expression of *DkPK7* (A)** and *DkPK8* **(B)** promoted *DkPDC* and *DkADH* transcript levels in persimmon leaves *in vivo.* Leaves infiltrated with *DKPK7* and *DKPK8* in ‘Mopanshi’ persimmon were sampled at 8 days after agroinfiltration. pMV2-GFP indicates the empty vector. *Error bars* represent the standard deviation (*n* = 12) (*p* < 0.05).

Previous studies have reported that acetaldehyde-related *DkPDC* and *DkADH* genes act as key participants in natural deastringency in C-PCNA persimmon (Mo et al., 2016). The PDC enzyme plays an important role in pyruvate metabolism, which is capable of catalyzing pyruvate into acetaldehyde. ADH catalyzes the conversion between acetaldehyde and ethanol. As downstream genes of *PK*, *DkPDC*, and *DkADH* (Min et al., 2012) expression levels were assessed by qRT-PCR in transiently over-expressing leaves (**Figure 8**). In the persimmon leaves infiltrated with *DkPK7*, the expression of five *DkPDC* and three *DkADH* genes were all increased; in particular, three *DkPDC* (*DkPDC2-4*) and two *DkADH* (*DkADH1-2*) genes exhibited significant differences in mRNA expression compared with the control (**Figure 8**). Among these genes, *DkPDC3* and *DkADH1* were expressed at higher levels, with increases of 2.5- and 2.6-fold, respectively. In the persimmon leaves infiltrated with *DkPK8*, *DkPDC1*, and *DkADH1* showed the highest mRNA levels with 3- and 2.8-fold increases, respectively. The transcript level of all *DkPDC*s and *DkADH*s were also increased in the persimmon leaves of the over-expressed *DkPK8* line, exhibiting a similar pattern as that in the over-expressed *DkPK7* line. Moreover, four *DkPDC* (*DkPDC1-4*) and two *DkADH* (*DkADH1, DkADH3*) genes had significant differences in mRNA expression compared with the control. These results demonstrated that the transient over-expression of both *DkPK7* and *DkPK8* in persimmon leaves can lead to the general up-regulation of the *DkPDC* and *DkADH* genes, which potentially facilitate the generation of acetaldehyde and lead to natural deastringency in C-PCNA persimmon.

## Discussion

Pyruvate kinase catalyzes the irreversible conversion of PEP and ADP to pyruvate and ATP, which is a primary control site of glycolysis and acetaldehyde metabolism in plants (Plaxton, 1996). Numerous *PK* family genes have been characterized and shown to be important regulators of plant growth, including a range of biosynthetic pathways as well as respiration (Knowles et al., 1998; Ambasht and Kayastha, 2002; Oliver et al., 2008). Previous studies have reported that PKc plays a potential regulatory role in loquat and banana fruit ripening (Law and Plaxton, 1997; Qin et al., 2013), and we recently found that *DkPK1* might be involved in the natural loss of astringency during the last developmental stage in persimmon (Guan et al., 2016). Thus, isolating and investigating the roles of other *PK* family genes in C-PCNA persimmon fruit should contribute not only to the functional analysis of the *DkPKs* but also to determining the mechanism of astringency removal in C-PCNA cultivars.

In our previous work, the full-length cDNAs of the *DkPK1-6* genes were isolated, and the *DkPK1* expression pattern correlated well with a decrease in the soluble PA content during the last C-PCNA persimmon developmental stage (Guan et al., 2016). In the present study, we obtained the transcripts for eight novel *DkPK* genes (*DkPK7-14*) using RACE-PCR and gene cloning approaches. Furthermore, the transcript levels of *DkPK7* and *DkPK8* appeared to be related to soluble or insoluble PA content during the fruit developmental process in C-PCNA persimmon in the same way as *DkPK1*. *DkPK1* expression in C-PCNA persimmon was negatively correlated with soluble PA content during the last stage (Guan et al., 2016). The *DkPK7* expression pattern showed a similar correlation with the change in insoluble PAs in C-PCNA persimmon throughout development (**Figures 1C** and **4**). *DkPK8* expression in the C-PCNA type increased remarkably during the last stage and displayed the opposite tendency to soluble PA content during the same stage (**Figures 1C** and **4**). In addition, *DkPK7* and *DkPK8* were closely clustered together with *DkPK1*, and all belong to the cytosolic-1 subfamily based on the phylogenetic analysis (**Figure 2**). Both cytosolic (PKc) and plastid (PKp) isozymes exist in plants, and these isoenzymes show clear differences in their respective physical and regulatory characteristics (Plaxton, 1996; Plaxton et al., 2002). PKp is critical for several plastidic biosynthetic pathways and are more likely related to the accumulation of oil in plant seeds (Knowles et al., 1998; Andre et al., 2007; Oliver et al., 2008). In contrast, PKc is involved in various biosynthetic pathways, including respiration and acetaldehyde metabolism (Knowles et al., 1998; Oliver et al., 2008). Generally, PKc is more likely to be involved in regulating fruit ripening (Law and Plaxton, 1997; Qin et al., 2013). Furthermore, in our study, the subcellular localization of DkPK agreed with the cluster analysis that both DkPK7 and DkPK8 belong to the cytosolic family of proteins (**Figures 2** and **5**). These results are also consistent with previous reports that DkPK1 was located in the cytoplasm of tobacco leaves. Thus, based on the expression analysis and protein subcellular location, *DkPK7* and *DkPK8* are more likely to be associated with acetaldehyde metabolism, which leads to the natural removal of astringency in C-PCNA persimmon.

The acetaldehyde-mediated coagulation effect is thought to be the principal cause of astringency removal not only in non-PCNA persimmon but also in C-PCNA types (Taira, 1995; Zhang et al., 2012; Mo et al., 2016). Non-PCNA persimmon requires deastringency treatment before eating because its astringency cannot be removed naturally on the tree; treatment with CO_2_ or ethanol accelerates the accumulation of acetaldehyde, which is directly involved in PA coagulation to convert soluble PAs into insoluble PAs (Taira, 1995; Salvador et al., 2007). In the C-PCNA type, the astringency can be removed naturally on the tree, mainly because of the coagulation of soluble PAs during the last developmental stage of persimmon fruit (Zhang et al., 2012; Guan et al., 2016; Mo et al., 2016). PK, the upstream enzyme in the glycolytic pathway, plays an important role in acetaldehyde metabolism. It may be a key gene in the processes of both artificial and natural deastringency. In our previous study, the *DkPK* transcript was abundantly altered during artificial deastringency; the transcript was significantly enriched among the differentially expressed genes of ‘Luotian-tianshi’ persimmon treated with 5% ethanol and was remarkably up-regulated in ‘Eshi 1’ persimmon fruit exposed to 40°C water (Luo et al., 2014; Guan et al., 2016). Moreover, *DkPK1* characterization indicated that it might facilitate the natural loss of astringency in C-PCNA-type fruit during the last developmental stage (Guan et al., 2016). In the present study, for further rapid gene functional analysis, the transient transformation technique was performed in persimmon leaves *in vivo*. Transient over-expression of both *DkPK7* and *DkPK8* in persimmon leaves resulted in a significant decrease in soluble PA content (**Figure 7**) and an increase in the expression of both *PDC* and *ADH* genes as a whole (**Figure 8**); these changes were closely related to acetaldehyde metabolism.

Pyruvate decarboxylase (*PDC*), a gene downstream of *PK*, catalyzes the formation of acetaldehyde from pyruvate, and ADH catalyzes the reversible reaction between acetaldehyde and ethanol (Strommer, 2011). These two genes have recently been characterized as key elements involved in natural astringency loss in C-PCNA persimmon (Mo et al., 2016). In our present study, significant increases in the levels of transcripts of *DkPDC2, DkPDC3*, and *DkPDC4* were observed after over-expressing *DkPK7*, and increases in *DkPDC1, DkPDC2, DkPDC3*, and *DkPDC4* were observed after over-expressing *DkPK8* in persimmon leaves. Furthermore, significant increases in *DkADH1* and *DkADH2* transcript were observed after over-expressing *DkPK7*, and an increase in *DkADH1* and *DkADH3* transcripts was also obtained in persimmon leaves transformed with *DkPK8* (**Figure 8**). The relatively constant expression of individual genes in the over-expressing leaves might be because of the complex genetic characteristics of allohexaploidy. Those results agree with the previous report that the up-regulation of *ADH* and *PDC* can be stimulated by the over-expression of *DkPK1* in persimmon leaves (Guan et al., 2016). The up-regulated expression of *DkPK*, *DkPDC*, and *DkADH* genes that is closely related to aldehyde metabolism often is thought to promote the generation of acetaldehyde. For example, abundant *DkADH* and *DkPDC* transcripts are often observed together with acetaldehyde accumulation during the process of artificial deastringency (Min et al., 2012; Mo et al., 2016). It is worth noting that insoluble PAs showed an increase in leaves infiltrated with *DkPK7* and *DkPK8*, and soluble PAs decreased at the same time (**Figure 7**). It has been reported that increasing insoluble PAs during artificial deastringency is due to the coagulation of soluble PAs (Taira, 1995; Salvador et al., 2007). Moreover, Mo et al. (2016) reported that up-regulation of the *DkPDC* and *DkADH* genes is involved in the decrease in soluble PAs during the last developmental stage in C-PCNA persimmon. Thus, the up-regulation of both *DkPDC* and *DkADH* in *DkPK*-over-expressing leaves might promote the synthesis of acetaldehyde, which reacts with soluble PA, resulting in an increase in insoluble PA.

In addition to the flesh, the up-regulation of *DkPK7* and *DkPK8* in seeds might contribute to natural deastringency in C-PCNA persimmon. Previous studies have reported that the seeds of some non-PCNA and C-PCNA persimmon can accumulate a volume of volatile acetaldehyde, which accelerates the coagulation of soluble PAs (Sugiura and Tomana, 1983; Mo et al., 2016). Mo et al. (2016) reported that the acetaldehyde accumulation in ‘Luotian-tianshi’ seeds showed a rapid rise during the late development stage; this increase corresponded well with the expression patterns of *DkADH* and *DkPDC* observed in the seeds. In this study, the expression of *DkPK7* and *DkPK8* in ‘Luotian-tianshi’ seeds were higher among the different tissues (**Figure 6**); this increase not only correlates with changes in acetaldehyde concentration in seeds but is also consistent with the analysis results for *DkPK1* tissue-specific expression (Guan et al., 2016; Mo et al., 2016). Thus, we presume that the accumulation of acetaldehyde in seeds could promote the natural loss of astringency in ‘Luotian-tianshi’ persimmon fruits.

In summary, based on RNA-Seq and EST data, eight novel *DkPK* family genes were obtained using RACE-PCR and a homology cloning strategy. Both *DkPK7* and *DkPK8* exhibited an increase in transcript levels during the last developmental stage in C-PCNA persimmon that was negatively correlated with a reduction in the level of soluble PA. Both the phylogenetic analysis and subcellular localization further confirmed that *DkPK7* and *DkPK8* are cytosolic proteins. Furthermore, transient over-expression of *DkPK7* and *DkPK8* in persimmon leaves *in vivo* significantly reduced the level of soluble PAs and increased the content of insoluble PAs. At the same time, the expression of the acetaldehyde-related genes *DkPDC* and *DkADH* also increased significantly. Taken together, we suggest that the *DkPK* genes are involved in the natural loss of astringency in C-PCNA persimmon via enhancement of the transcript levels of the *DkPDC* and *DkADH* genes. Our findings contribute to the functional analysis of *DkPK* family numbers in plants and pave the way for elucidating the mechanism of deastringency in C-PCNA persimmon.

## Author Contributions

YY, XD and CG conceived and designed the experiments. CG performed the experiments. YY and CG participated in the data analysis and wrote the manuscript. ZL, FM, QZ, and XD helped to draft and review the manuscript. All authors read and approved the final manuscript.

## Conflict of Interest Statement

The authors declare that the research was conducted in the absence of any commercial or financial relationships that could be construed as a potential conflict of interest. The reviewer HZ declared a shared affiliation, though no other collaboration, with several of the authors CG, QZ, ZL to the handling Editor, who ensured that the process nevertheless met the standards of a fair and objective review.

## Footnotes

1 http://linux1.softberry.com/

2 http://meme-suite.org/tools/meme

3 http://www.genome.jp/tools/motif/

## Supplementary Material

FIGURE S1 **Multiple sequence alignment of the deduced DkPK amino acid sequences**. The deduced amino acid sequences of DkPK1-6 were obtained from Guan et al. (2016). The multiple sequence alignment was performed with DNAMAN 6.0.
