# Supplementary material for: DkPK Genes Promote Natural Deastringency in C-PCNA Persimmon by Up-regulating DkPDC and DkADH Expression
Source: Front Plant Sci. 2017 Feb 13;8:149. doi: 10.3389/fpls.2017.00149 (PMC5303730; doi:10.3389/fpls.2017.00149)
Supplement: FIGURE S1 — Multiple sequence alignment of the deduced DkPK amino acid sequences. The deduced amino acid sequences of DkPK1-6 were obtained from Guan et al. (2016). The multiple sequence alignment was performed with DNAMAN 6.0. [file Table_1.DOC]

**Table S1. List of primer sequences used in this article.**

| **Purpose** | **Name** | **Primary PCR (5' to 3')** | **Secondary PCR (5' to 3')** |
| --- | --- | --- | --- |
| 3'RACE | *DkPK7* | GATGCCTTCACCTTAATGGCAGCT | TTGGTTCTCCAACGTATTTGACAG |
| 5'RACE | *DkPK7* | GTGCAACATGGCTGGAAAACCTG | AAATCTTAGACCAACACGTGCAGA |
| 3'RACE | *DkPK8* | TGCGAAGATCGAAAATTTCGAGGG | ATTGATCTCCCACCAGAGAAGGTAT |
| 5'RACE | *DkPK8* | CCAGTAAATGTCCAGCGGAGTTGA | AGCTCTTCCTGTCGAAGTGAAGCAA |
| 3'RACE | *DkPK9* | TTCTGGCAAATCTTTAGTATCCTT | TGACAGATAAATTAGTGGGTGGGG |
| 5'RACE | *DkPK9* | AATGTAATGAGGAAAAAGGCATCC | TCATTCGTATCAACTGTGCACATGG |
| 3'RACE | *DkPK10* | CAGACAGTATTTCGGAGGAAATCT | TCCCTTCTGTCACGTTCTCGACCTG |
| 5'RACE | *DkPK10* | GCCCGAGGGAGTAAAAGTCCAGG | ATGCCACCATCCACAAGGAGTTCAT |
| Full-length cDNA cloning | *DkPK7* | TTTCCATGGGTAGCATTATAT | AGGCACCATGACAGGAGTGC |
|  | *DkPK8* | TGGCGGGAAGGAGAATAGC | CAGGCGCACCACGGCACAG |
|  | *DkPK 9* | TATAAATTGAAAGAAGAAA | CTCTTTAAACATCAATCGC |
|  | *DkPK10* | CCTGCTGTGGGTTGTATCGC | CTTGCTGGTCCGTGGGAAT |
|  | *DkPK 11* | CGATCGGTGGATTCAATTC | TTTCACCGCTCAGCATTAC |
|  | *DkPK12* | GCATGGCATTACGTTCCCG | CACCGCCTCCAACTGTCTC |
|  | *DkPK 13* | GCATGGCATTACGTTCCCGC | GAGACAGTTGGAGGCGGTG |
|  | *DkPK14* | GCGCTTCGGCGGTTGGAG | CAACAATAATAGATCCACG |
| Real-time  PCR | *DkPK7* | TGGAGAGTGAACAATGAGC | CAAAGGCAGTGAAAAAGG |
|  | *DkPK8* | GGTTTGACTTTTCATGGG | AGGGGAATAGAACGCTCT |
|  | *DkPK 9* | TCTGCCTTCACCTTTGTA | CTATTTGTAGTGCTGCCC |
|  | *DkPK10* | TCGTGATAGTGATGTTGCT | GGTGTATTTAATTGCCTGC |
|  | *DkPK 11* | GCTCGCTTCAACTTCTCTC | ATCATGTTCTCATCACCCT |
|  | *DkPK12* | CGACCACCACTTGTAATCC | ACTCCCACTCAAAACCCTA |
|  | *DkPK 13* | TTGTTGGCCTATACTTAGC | ATACCCTTTCTCCCTTTG |
|  | *DkPK14* | CCTCTCACTGTCATATACTCG | ATGCCATCTGCTTCTTGT |
| Over-expression vector | PMV2-DkPK7 | CGGGTACCATGCACGCGAATCATCTTCT | GCTCTAGACTAATCTTCAAGCTCAATGA |
|  | PMV2-DkPK8 | CGGGTACCATGCACTCCAGTAACTTGCT | GCTCTAGACTAATCTTCAAGCTCGATAA |
| Subcellular localization | 101LYFP-DkPK7 | CGGGATCCATGCACGCGAATCATCTTCT | GGGGTACCATCTTCAAGCTCAATGATCT |
|  | 101LYFP-DkPK8 | CGGGATCCATGCACTCCAGTAACTTGCT | GGGGTACCATCTTCAAGCTCGATAATCT |
