# Supplementary figures and images for: DkPK Genes Promote Natural Deastringency in C-PCNA Persimmon by Up-regulating DkPDC and DkADH Expression
Source: Front Plant Sci. 2017 Feb 13;8:149. doi: 10.3389/fpls.2017.00149 (PMC5303730; doi:10.3389/fpls.2017.00149)

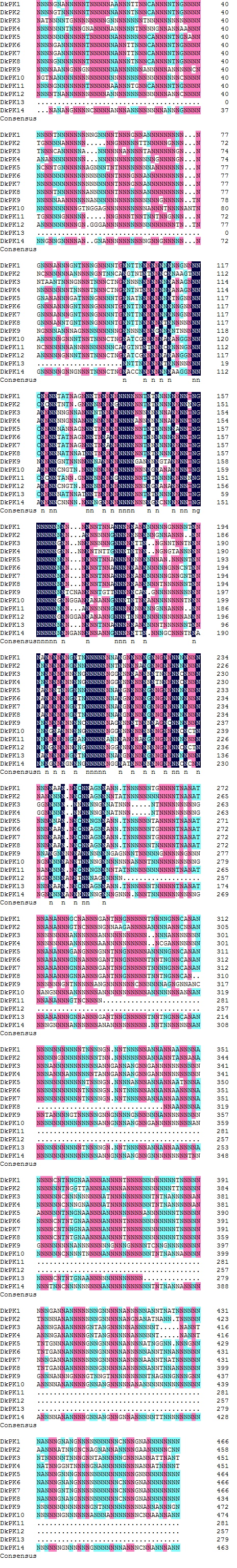

Supplement: Supplementary file 2 [file Image_1.JPEG]
